# Small extracellular vesicles purification and scale-up

**DOI:** 10.3389/fimmu.2024.1344681

**Published:** 2024-02-26

**Authors:** Xinya Zheng, Hongru Ai, Kewen Qian, Guangyao Li, Shuyi Zhang, Yitan Zou, Changhai Lei, Wenyan Fu, Shi Hu

**Affiliations:** ^1^Department of Biomedical Engineering, College of Basic Medical Sciences, Second Military Medical University, Shanghai, China; ^2^School of Gongli Hospital Medical Technology, University of Shanghai for Science and Technology, Shanghai, China; ^3^Department of Biophysics, College of Basic Medical Sciences, Second Military Medical University, Shanghai, China; ^4^Department of Assisted Reproduction, Shanghai Ninth People’s Hospital, Shanghai Jiao Tong University School of Medicine, Shanghai, China; ^5^Fahe Life Science and Technology Inc., Shanghai, China

**Keywords:** small extracellular vesicles, purification, scale-up, industrialization, therapeutics

## Abstract

Exosomes are small extracellular vesicles (sEVs) secreted by cells. With advances in the study of sEVs, they have shown great potential in the diagnosis and treatment of disease. However, sEV therapy usually requires a certain dose and purity of sEVs to achieve the therapeutic effect, but the existing sEV purification technology exists in the form of low yield, low purity, time-consuming, complex operation and many other problems, which greatly limits the application of sEVs. Therefore, how to obtain high-purity and high-quality sEVs quickly and efficiently, and make them realize large-scale production is a major problem in current sEV research. This paper discusses how to improve the purity and yield of sEVs from the whole production process of sEVs, including the upstream cell line selection and cell culture process, to the downstream isolation and purification, quality testing and the final storage technology.

## 1 Introduction

sEVs are nanoscale vesicles secreted by cells with a lipid bilayer membrane structure. It was first discovered in 1983 in sheep reticulocytes in culture and was named “exosome” in 1987 (1). sEVs are generally considered to be between 30-150 nm in size (2). At first people considered cellular metabolic wastes and not taken seriously. sEVs are widely found in almost all tissue cells and body fluids (3) and are rich in lipids, proteins, and nucleic acids (4). sEVs can travel between cells and carry a wide range of substances from the parent cell for intercellular communication (5), playing an irreplaceable role in physiological and pathological situations. Compared to traditional stem cell therapies, the small size of sEVs makes it easier for them to be endocytosed by cells to transfer their cargo to recipient cells, and because sEVs are less immunogenic, they can be administered repeatedly (6).

sEVs are widely used in biomedicine in three main directions. The first is the extraction of sEVs in the pathologic microenvironment as biomarkers for disease diagnosis (7). The second is that small extracellular vesicles themselves contain a variety of cytokines, proteins, messenger RNAs (mRNAs), microRNAs (miRNA), long non-coding RNAs (IncRNAs), lipids, metabolites and even DNA fragments (8), that produce therapeutic effects (9). The third is the ability of sEVs to transport drugs (10). However, due to current technological limitations, it remains a major challenge to obtain high purity, high quality and sufficient doses of sEVs for clinical use.

sEVs were demonstrated to play vital roles in intercellular communication in normal physiological processes and in the pathogenesis of disease, including cancer neurodegenerative, diseases and cardiovascular diseases. sEVs of different origins have different roles to play (11). For example, sEVs of immune cell origin fight disease primarily by promoting immunity, whereas sEVs of stem cell origin promote tissue regeneration. In addition, due to the different purity and activity of sEVs obtained from different isolation methods, the results of their use in disease therapy are not exactly the same (12). The efficacy of sEVs obtained by immunoaffinity capture may not be as good as those obtained by other isolation methods due to the difficulty of removing the antibodies used for capture. Size exclusion chromatography is gentle, and the sEVs obtained are more pure and active, and are used for better results in disease treatment.

The purity and yield of sEVs are affected by multiple conditions, mainly the choice of cell line (sample sources), cell culture, isolation techniques, storage, etc.

## 2 Sample selection and cultivation methods

Different samples contained different sEV content and isoforms. Different cells and cultures produce different sEVs.

### 2.1 sEV-producing cells and MSCs source selection

The vast majority of cells in the body can produce sEVs (13), which are found throughout our body. The sources of sEVs are mainly divided into two main categories, one is the direct extraction of sEVs from body fluids secreted by the human body, such as serum (14), lymph, cerebrospinal fluid (15), bile (16), plasma (17), urine (18), breast milk (19), saliva, etc., and the other is the extraction of sEVs from the supernatants of a variety of cell culture media (20). However, since natural body fluids, especially plasma, contain cellular debris, apoptotic vesicles, microvesicles, and plasma proteins, which are not easily separated from sEVs due to their overlapping sizes and biochemical properties, resulting in a low purity of the isolated sEVs (21). The urine has fewer interfering particles than the plasma, but a low concentration of sEVs (21). It’s obvious sEVs obtained from urine are more pure, but because of their low concentration, they require a larger volume than in plasma extraction to obtain the same mass.

In contrast, *in vitro* cell culture supernatants are easier to obtain and the quality of sEVs obtained is more stable, so most of the existing techniques are extracted and isolated from conditioned cell cultures (22, 23). Stem cells with high productivity have been used for the longest time for *in vitro* cell culture, with mesenchymal stem cells (MSCs) being the most used (24). Depending on the source, MSCs can be categorized into bone marrow MSCs, adipose MSCs, human umbilical MSCs, dental pulp MSCs and so on (25). Stem cells from different sources proliferate at different rates on their own and produce sEVs of varying quantity and quality (26), among which human umbilical MSCs produce the largest number of sEVs and the largest size (27), meanwhile, human umbilical MSCs are able to be stably cultured in serum-free medium (28), which effectively avoids the contamination by the impurities in serum in the subsequent isolation process, and is conducive to the large-scale production of sEVs.

### 2.2 Cell culture

The occurrence and secretion of cellular sEVs are influenced by multiple conditions. On the one hand, small molecules can influence sEV production and secretion. For example, thrombin pretreatment enhances the ability of MSCs to produce extracellular vesicles and the quality of extracellular vesicles is not affected (29). Adiponectin stimulates the production and secretion of sEVs by binding to T-cadherin on MSCs (30). N-methyldopa and norepinephrine can triple the sEV production of MSCs (31). Melatonin-treated sEVs enhance the regenerative potential of MSCs (32). In addition, external environments such as magnetic field (33), flow and stretch (34), ultrasound (35), PH (36), hypoxia (37), temperature (38), and light (39) affect the synthesis and release of sEVs.

To further obtain higher yields and quality of sEVs, Three dimensional culture systems are being used for sEVs production. The three dimensional culture improves sEVs production by increasing cell-cell-cell matrix interactions (40). Common three dimensional culture methods are hanging droplet culture and microporous array method, the hanging droplet culture is simple and easy to execute, but the yield is limited and there are limitations for sphere size adjustment, so mass production with hanging droplet culture will be time-consuming (41). The microporous array method inoculates cells into a series of small wells to which cell growth-promoting materials can be added to promote cell proliferation and sEV synthesis, In addition, the microporous array method is easier to produce three dimensional spheres than the droplet method and has a higher throughput (42), which means microporous array method more suitable for mass production. In addition, artificial sEVs with low cost, high yield and stable quality are expected to be a powerful alternative to natural sEVs (43).

## 3 sEV isolation and purification

### 3.1 Common isolation techniques

Common isolation techniques include ultracentrifugation, ultrafiltration, immunoaffinity capture, size exclusion chromatography and precipitation.

Ultracentrifugation is the classical method for sEV isolation (44). At the same time, it is also the most widely used isolation technique (45). Ultracentrifugation is based on the separation of sEVs and impurities in the samples with different densities and sizes (46), the dead cells, cellular debris, and large extracellular vesicles in the samples are successively removed by different rotational speeds, and finally obtain the sEVs in the supernatant (45). Ultracentrifugation can process a large number of samples at one time and is easy to perform, but produces less pure and time-consuming sEVs because of the different subtypes of sEVs have overlapping density ranges (47). Formation of a density gradient medium with sucrose or iodixanol in combination with ultracentrifugation improves the purity of sEVs, but prolonged incubation with high sucrose concentrations impairs the structure of sEVs (48). In addition, the polymer density layer is expensive and not suitable for sEV scale-up.

Ultrafiltration is based on the isolation of different extracellular vesicles with different sizes, which can only pass through a series of semi-permeable membranes with defined pore sizes (49). However, since extracellular vesicles are deformable, vesicles that do not conform to the size can also be deformed to pass through the pore resulting in sEV impurity, so they are only used for preliminary isolation (50).

Immunoaffinity capture is an sEV isolation technique based on antigen-antibody interactions, in which immobilization of a specific antibody allows for specific binding of an antigen unique to the surface of the sEV, and thus capturing the sEVs (51). However, the overlap of antigens between different subpopulations and the difficulty of removing capture antibodies can affect later functional assay studies of sEVs, that not conducive to subsequent sEV research and applications.

Size exclusion chromatography (SEC) is also an isolation method based on sEV size, where large particles are unable to enter the gel pores and small sEVs are allowed to enter, which is a milder separation method that yields sEVs with higher purity and activity (52). However, the resolution of SEC decreases when the particles are close to or larger than the upper limit of the pore size, and so SEC is often used in conjunction with ultracentrifugation (53) and ultrafiltration (54) to improve the purity of sEVs.

Precipitation is sEVs under the action of polyethylene glycol usually, the solubility decreases leading to the precipitation of sEVs, and then sEVs can be obtained by low-speed centrifugation (55), which is easy to operate, does not require special equipment, and is conducive to the preparation of large-scale, but is prone to the introduction of impurities leading to the sEVs are not high purity (56).

### 3.2 sEVs isolation kits

As the demand for sEVs increased, a variety of commercial kits rapidly emerged. Kits principle is based on traditional sEV separation methods such as ultrafiltration, sedimentation and so on. Commercial kits do not require special equipment, with the advantages of simple operation and short time consuming, and can isolate sEVs from most common body fluids and cell culture supernatants (57). However, kits are expensive and cannot process a large number of samples at once, so they are not suitable for high-volume processing. Furthermore here are significant differences in the purity and quantity of sEVs collected by different kits (47). For example, the yield of sEVs obtained with the invitrogen kit is dozens of times more than that obtained with conventional ultracentrifugation, but the purity of the output sEVs is unsatisfactory, and the sEV isolation kit requires pre-separation before use, which makes the experimental process cumbersome (58).

### 3.3 Emerging sEVs isolation technologies

Although there are many techniques for sEV isolation and purification, all of the above techniques have significant drawbacks and are not suitable for large-scale production of sEVs (**Table 1**). Microfluidics is an emerging method for sEV isolation, which is a technique for controlling fluids in micrometer-sized channels that relies on a range of sEV properties including immunoaffinity, density, and size, and it overcomes the limitations of traditional methods by offering advantages such as low cost, small size, speed, sensitivity, labeling-free, and high recoveries (57). It is expected to replace traditional methods in the future and play an important role in the industrialized mass production of sEVs in the future.

**Table 1:** Comparison of different isolation techniques.

Classification	Isolation technique	Process time		Advantages	Disadvantages	References
Centrifugation techniques	Ultracentrifugation	3-6h		Simplicity of operator Single processing sample is large Most commonly used separation techniques	Low purity Time-consuming	(45, 47, 48, 59)
Size-based techniques	Density gradient centrifugation	24h		Higher purity compared to differential centrifuges	Long incubation time leads to destruction of sEV	(48, 60, 61)
	Ultrafiltration	1-3h		Wide range of application Suitable for primary screening	Low purity Not suitable for plasma	(49, 50)
Capture-based techniques	Size exclusion chromatography	0.5-2h		The obtained exosomes have high activity High purity	Complicated operation High cost and expensive instruments	(52–54)
	Magnetic beads and immunoaffinity capture	4h		High purity High resolution high recoveries	Low yield Bound antibodies are not easily removed	(48, 51, 62, 63)
Polymer-based techniques	Commercial kits	0.5-3h		No special equipment required Easy operation Short time-consuming	Low production High cost Laboratory only	(47, 57 ,58)
Microfluidics-based techniques	Size-based microfluidics	0.5-1h		Label-free, fast, highly reproducible, highly recoverable and high resolution	Not able to separate sEVs that have the same size	(64–66)
	Immunoaffinity-based microfluidic separation	0.5-1h		Low cost, small size, speed, sensitivity, labeling-free, and high recoveries	Bound antibodies are not easily removed	(57, 66, 67)
	Dynamic microfluidics	0.5-1h		High rate, purity Simple microchannel structure Controllable process	High demands on the manipulator	(66, 68–70)

The EXODUS system separates and purifies sEVs by two coupled oscillators generating a dual-frequency transverse wave on the membrane, which produces sEVs at a rate, purity, and throughput that are far superior to the others (68). Asymmetric-flow field-flow fractionation technology (AF4) is label-free, fast, highly reproducible, highly recoverable and high resolution, which helps to separate different sEV isoforms (64). However, because AF4 separates based on particle size, it is not able to separate sEVs that have the same size, but are actually different. Double tangential flow size screening-based microfluidic chip greatly improves sEV recovery rate and purity. Its sEV recovery rate up to 77.8, acquired sEVs can be directly used for sEV analysis (65). Capture of sEVs by altering temperature was devised by Kenichi Nagase (71).

Whether it is the traditional separation technology or the microfluidic technology in the last two years, it has not fully achieved the ideal separation effect. Based on the properties of sEVs and downstream applications, it may be a useful idea to combine different isolation methods to get better separation effect. The combination of different techniques may offer the possibility of efficiently obtaining high-purity and high-yield sEVs.

## 4 Quality testing and control of sEVs

Since the present technologies does not allow for a good isolation of sEVs from other impurities, the subsequent assessment of the purity and quality of the sEVs obtained is particularly important. Different subtypes of sEVs contain different proteins, lipids, and nucleic acids because of the different cells of origin of the sEVs (72).

These differences of different sEVs have an important role in the assessment of sEV subtypes, such as the tetraspanins (CD9, CD63, CD81) that are often used to differentiate subpopulations of extracellular vesicles and to assess sEV purification (73). Commonly used techniques for sEV detection include nano-flow cytometry (74), flow cytometric analysis (75), ELISA (76), transmission electron microscopy and so on. These techniques are used to assess the quantity and purity of sEVs in samples. To provide a better platform for the use of sEVs in the clinic.

## 5 Storage of sEVs

From the current study, the storage conditions of sEVs including temperature, storage time and even the number of freezing and thawing cycles have a great impact on the concentration, purity and function of sEVs (77). Common storage conditions in the laboratory are 4°C, -20°C, and -80°C., the concentration and purity of sEVs decreases accordingly with increasing temperature and storage time. For commercial and clinical use, long-term storage of sEVs is generally required. Because -80°C can effectively inhibits biologically active proteins and reduces the loss of sEVs, -80°C is usually considered to be the optimal temperature for sEV storage (78). However, this storage method usually makes sEVs susceptible to “frostbite”, mainly due to osmotic imbalance, so cryoprotectants are usually added during the freezing process to maintain protein stability and prevent osmotic damage (79). Commonly used cryoprotectants such as trehalose prevent aggregation by avoiding internal icing of sEVs, while the addition of trehalose contributes to the colloidal stability of sEVs (80). It has been shown that the addition of human albumin and trehalose during storage helps to improve long-term storage of sEVs, maintain freeze-thaw stability, and increase sEV recovery when diluents are used downstream (81).

However, -80°C is not suitable for the transportation and application of sEVs, and not all factories and laboratories have -80°C storage conditions. Therefore, freeze-drying and spray-drying are used for the preservation of sEVs, and studies have shown that freeze-drying can be used for long-term preservation at 4 °C (82), reducing storage requirements and costs for sEVs. In addition, repeated freezing and thawing can lead to a decrease in the number of sEVs and an increase in their size (83), so we should avoid repeated freezing and thawing process as much as possible to ensure the quality of sEVs during storage and transportation.

## 6 Laboratory and scale-up of sEVs

In the past few years, the clinical application of sEVs has become more and more widespread, however, in order to achieve significant clinical therapeutic effects require a certain dose of sEVs, but with the current production technology of sEVs, the production of sEVs is not high (84). With the increasing demand for sEVs, the traditional method of isolating sEVs from body fluids, such as human plasma, is obviously unable to meet the demand for the use of sEVs, so the large-scale production of sEVs is crucial. Less mature cells such as MSCs are often used in industry for culture to obtain sEVs (85). In reality, serum-free medium is usually used for stem cell culture due to the high content of endogenous extracellular vesicles in fetal bovine serum (86), which is not conducive to later isolation and purification. Studies show that switching from serum-containing to serum-free media produces sEVs that exhibit stronger therapeutic effects (87). However, it has been shown that the use of serum-free media leads to an increase in reactive oxygen species and emergency-related proteins (88). However, it is undeniable that serum-free medium it is favorable to improve the purity of sEVs and reduce the unknown side effects of sEVs during clinical application.

In addition sEV production is related to the surface area to which cells can attach in the bioreactor. Therefore microcarriers are particularly important in bioreactors, which are generally spherical in shape to provide a larger value-added area for adhesion (89). A variety of bioreactor systems are used for large-scale production, such as hollow-fiber membranes (90), three-dimensional stirred-tank bioreactors (91).

**Figure 1:**
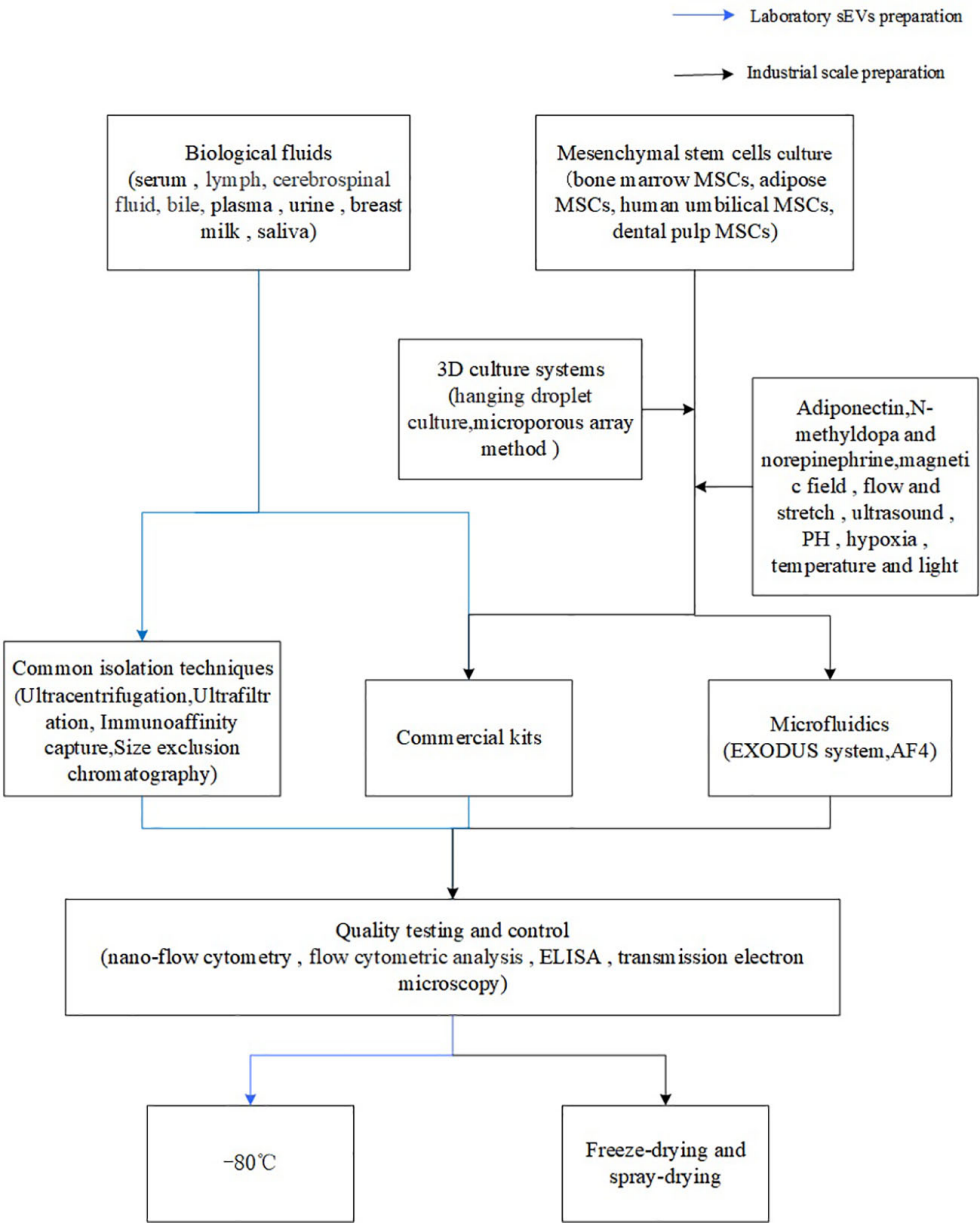
Flowchart of sEVs preparation.

**Figure 2:**
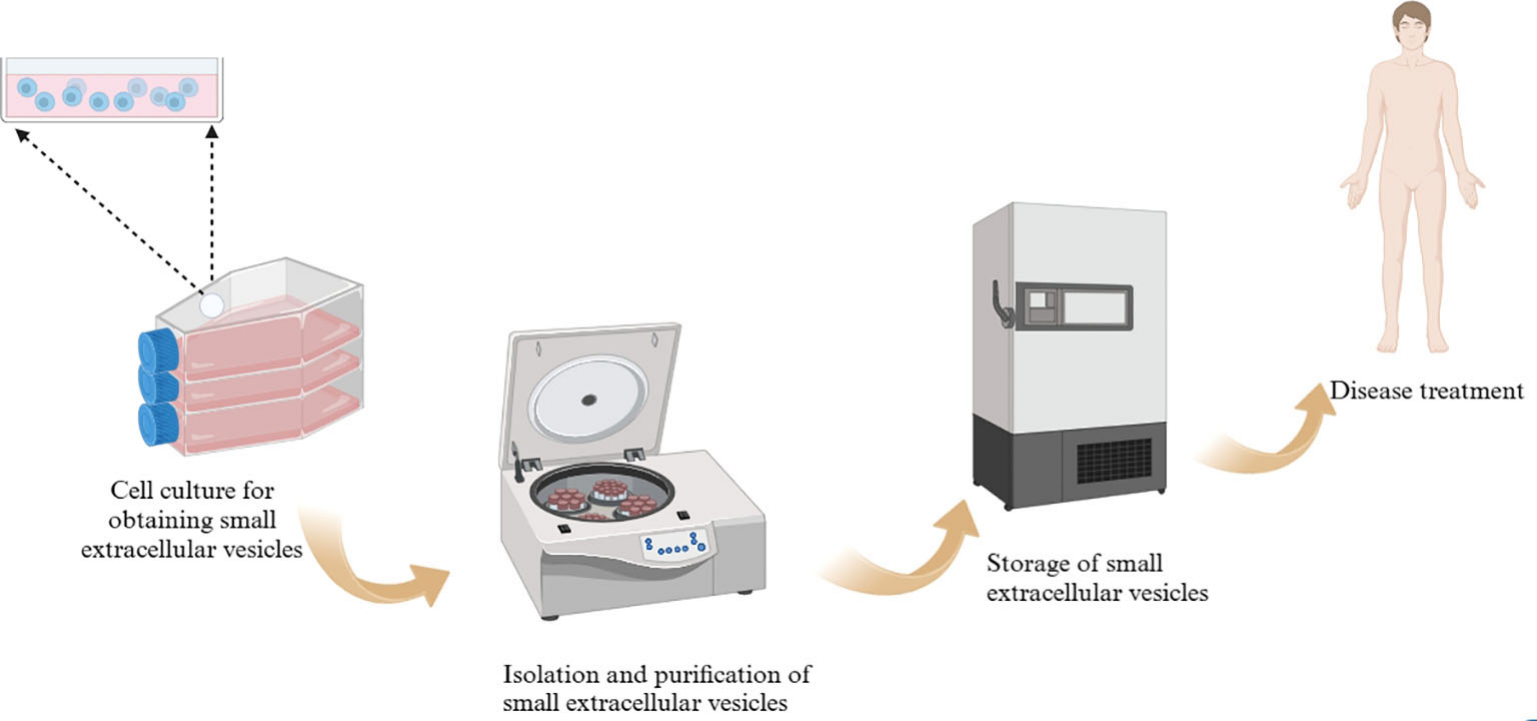
sEVs preparation process.

Of course, the production of sEVs from the general laboratory to industrial mass production is not a simple process, which not only involves the selection of cell lines in the early stage, cell culture, but also includes the isolation and purification of the latter, quality control, storage (**Figures 1**, **2**). And importantly tighter control of lot-to-lot consistency of sEVs is not easy (92). There is still a long way to go for sEV purification and scale-up.

## 7 Discussion

sEVs have a wide range of applications and are currently used in the diagnosis and treatment of diseases, cosmetic skincare and scalp care for hair regrowth. In particular, stem cell-derived sEVs show great potential for clinical therapy. sEVs can travel between cells for the purpose of intercellular communication. In addition, due to the small size of sEVs, it is easier to transfer cargo to recipient cells by endocytosis, and the low immunogenicity allows repeated administration. These properties determine that sEVs are more likely to produce good therapeutic results.

Despite the widespread use of sEVs, their use is limited in several ways. Currently, a major challenge in the field of sEV research focuses on the isolation and purification of sEVs and how to achieve large-scale production to meet the needs of society. Due to technological limitations, various methods currently have drawbacks, initially people tried to build on their strengths and avoid their weaknesses by combining different isolation methods, and then emerging technologies such as microfluidics were invented and incorporated into the existing isolation techniques, which resulted in an effective improvement of sEV purity and yield. In addition, with the use of advanced technologies such as serum-free media and bioreactors for sEV production, the yield of sEVs has been effectively increased, but nevertheless, there are still many challenges in the large-scale production of sEVs.

## Author contributions

SH: Writing – review & editing. XZ: Writing – original draft. HA: Writing – review & editing. KQ: Writing – review & editing. GL: Writing – review & editing. SZ: Writing – review & editing. YZ: Writing – review & editing. WF: Writing – review & editing. CL: Writing – review & editing.
